# Programmed Death-Ligand 1 (PD-L1) Positivity and Factors Associated with Poor Prognosis in Patients with Gastric Cancer: An Umbrella Meta-Analysis

**DOI:** 10.7759/cureus.23845

**Published:** 2022-04-05

**Authors:** Gashaw Hassen, Amita Kasar, Nidhi Jain, Shivankshi Berry, Jhanvi Dave, Michlene Zouetr, V Lakshmi N Priyanka Ganapathiraju, Tejaswini Kurapati, Stephanie Oshai, Mohamed Saad, Jahangirkhan Pathan, Sheetal Kamat, Raghavendra Tirupathi, Urvish K Patel, Rishabh K Rana

**Affiliations:** 1 Progressive Care, Mercy Medical Center, Baltimore, USA; 2 Medicine and Surgery, Parma University, Parma, ITA; 3 Medicine, Addis Ababa University, Addis Ababa, ETH; 4 Internal Medicine, Krishna Institute of Medical Sciences, Secunderabad, IND; 5 Medicine and Surgery, Himalayan Institute of Medical Sciences, Dehradun, IND; 6 Hematology and Oncology, Brooklyn Cancer Care, Brooklyn, USA; 7 Internal Medicine, Sir Ganga Ram Hospital, New Delhi, IND; 8 Internal Medicine, Dayanand Medical College and Hospital, Ludhiana, IND; 9 Internal Medicine, Nassau University Medical Center, New York, USA; 10 Internal Medicine, B.J. Medical College, Ahmedabad, IND; 11 Family Medicine, American Institute of Antigua College of Medicine, St John's, ATG; 12 Medicine and Surgery, Rangaraya Medical College, Kakinada, IND; 13 Medicine and Surgery, Narayana Medical College, Nellore, IND; 14 Medicine and Surgery, College of Medicine, University of Lagos, Lagos, NGA; 15 Gastroenterology, Theodor Bilharz Research Institute, Giza, EGY; 16 Internal Medicine, V. I. Vernadsky Crimean Federal University, Crimea, RUS; 17 Internal Medicine, Apollo Hospital, Bangalore, IND; 18 Internal Medicine, Keystone Health, Chambersburg, USA; 19 Public Health and Neurology, Icahn School of Medicine at Mount Sinai, New York, USA; 20 Preventive and Social Medicine/Community Medicine, Shahid Nirmal Mahto Medical College, Dhanbad, Dhanbad, IND

**Keywords:** overall survival (os), pd-l1, clinicopathological, tumor stage, lymph node metastasis, hazard ratio, poor prognosis, umbrella review, meta-analysis, gastric cancer

## Abstract

Gastric cancer (GC) is one of the most common malignancies throughout the world with late diagnosis and poor prognosis. The expression of programmed death-ligand 1 (PD-L1) in GC is attributed to immune evasion and tumor progression. PD-L1 positivity has both predictive and prognostic biomarker potential. Aiming to summarize a large amount of research and to provide a definitive conclusion to the conflicting results on the prognostic significance of PD-L1 expression in GC, we performed an umbrella review based on existing meta-analyses which were published recently (2016-2021) and indexed in the PubMed database. Preferred Reporting Items for Systematic Reviews and Meta-Analyses (PRISMA) guideline was used in August 2021 to screen articles, and data extraction with quality assessment was performed on the selected meta-analyses. Review Manager (RevMan) 5.3 software was used to analyze the HR and OR with a 95% confidence interval (CI) among PD-L1 positive GC patients. We also assessed the between-study heterogeneity (*I*^2^). Forest and Funnel plots were obtained, and a *P*-value of <0.05 was considered statistically significant. A total of 567 articles were screened, and we selected three meta-analyses with a total of 40 studies conducted over a period of 14 years. In our umbrella review, a total of 8,419 GC patients with an average PD-L1 positivity of 39% were analyzed. We found that PD-L1 positivity in GC patients is associated with poor prognosis (pooled HR =1.44, 95% CI: 1.24-1.68, *P*<0.00001) having higher mortality reducing the chances of overall survival (OS). However, there are no significant differences in PD-L1 expression among different lymph node (LN) metastases (OR=1.31, 95% CI: 0.98-1.74, *P*=0.07) and tumor, node, and metastasis (TNM) stages (OR=1.13, 95% CI: 0.80-1.58, *P*=0.50). Early identification of PD-L1 expression may help tailor cost-effective and targeted immunotherapy among GC patients. More research is needed to further understand how PD-L1 affects LN metastasis and tumor invasion.

## Introduction and background

Gastric cancer (GC) is one of the most common malignancies in the world, accounting for about one million cases each year and 5.7% of all cancer diagnoses. It has a dismal prognosis, as illustrated by the poor five-year survival rate and by the fact that most cases are already metastatic when they are identified [1,2]. The incidence of GC varies by up to 15-20-fold depending on where one lives [3,4]. Although the incidence of GC has decreased in recent decades, this decline is less pronounced in certain groups like the US white population [3].

*Helicobacter pylori* infection is one of the most well-known risk factors for GC. Other risk factors include gastroesophageal reflux disease, gastric ulcer disease, obesity, cigarette smoking, chemical exposure to high-temperature particulates such as asbestos or heavy metals, high-salt food, *N*-methyl-*N*-nitro-*N*-nitrosoguanidine, preserved meat, alcohol, gastric surgery, radiation exposure, Epstein-Barr virus, and inherited GSTM-1-null phenotype or CDH1(OMIM192090) gene mutation. GC is predominantly linked to the male sex, lower socioeconomic status, and certain races/ethnicities such as Asian/Pacific Islander, American Indian/Alaska Native, and Hispanic [5].

As more discoveries in the science of adaptive T-cell immunity emerge, it is now closely known how T-cells provide an immune response through interactions with inhibition and proliferation of cytokines. One of the prominent discoveries is the identification of programmed death-ligand 1 (PD-L1), a transmembrane protein that interacts with programmed cell death protein 1 (PD-1), a receptor on T-cells. The two have important roles in T-cell anergy, apoptosis, and death. Cytotoxic T-lymphocyte antigen 4 (CTLA-4) and PD-L1 were later established as immune checkpoints that keep normal cells from being attacked by T-cells [6]. The expression of immune-checkpoint molecules by tumor cells is also associated with tumoral immune evasion. In this regard, PD-L1 overexpression has been demonstrated in various types of cancers [7]. Over the years, PD-L1, also known as B7-homolog 1 (B7-H1), has been strongly established as a B7 family member involved in nullifying cell-mediated immunity [8].

PD-L1 is usually expressed in antigen-presenting cells such as dendritic cells, macrophages, and monocytes. PD-1/PD-L1 interactions contribute to the maintenance of peripheral tolerance of self-antigens in normal hosts [9]. Mounting evidence suggests that PD-L1 expression on solid tumors dampens antitumor T-cell responses. Moreover, PD-L1 tumor expression has been shown to correlate with different clinical outcomes in various solid malignancies. A recent study in Japan found that PD-L1 expression in patients with GC was directly related to a poor prognosis but, at the same time, would also be employed in providing breakthrough immunotherapy [9]. Other studies reported that PD-L1 expression was an independent prognostic factor after curative resection of GC [10]. Different published meta-analyses concluded with conflicting results regarding the correlation of PD-L1 presence in GC and its prognosis in terms of overall survival (OS), lymph node (LN) metastasis, and tumor staging.

Umbrella review is a topic-specific collection and evaluation of multiple systematic reviews and meta-analyses in systematic and comprehensive ways to provide a wider perspective of the evidence landscape on clinical outcomes [11]. It was first endorsed by *Cochrane* in 2009 and is considered the highest in the hierarchy of evidence evaluation, offering summary data with a broader view that fills the knowledge gap in the medical community [12,13]. Some of the advantages of umbrella review based on meta-analyses of observational studies and randomized controlled trials (RCTs) include: (a) synthesizing information on multiple exposure-outcome associations and (b) providing instructive multimodality for interventions [12,14].

Nowadays, umbrella review involves a cluster of reviews and meta-analyses, which integrates data on predictive and prognostic tests [15]. It also helps to conduct standardized methodological processes, which can assess the epidemiological credibility of the meta-analyses and their findings [16]. As there seem to be some gaps in understanding the prognostic significance of PD-L1 markers in GC, we decided to conduct an umbrella review to arrive at more solid evidence. Our study will also address additional gaps that exist in terms of generalizability of previous studies and lack of collating multiple meta-analyses.

It is also worth mentioning that despite being at the top of the evidence hierarchy, umbrella review by itself is not free from imperfection. There are some flaws and drawbacks associated with umbrella review as a result of the very nature of either the meta-analyses or the individual primary studies included within the meta-analyses. Some of these undesirable qualities may include (i) observational study-related bias and uncertainty and (2) meta-analysis-related narrower literature search, moderate qualities of included studies, and reporting on selective outcomes only [17].

## Review

Methodology

Endpoint

The primary aim of our study was to evaluate the prognostic significance of PD-L1 when it is present in GC patients in terms of survival rate while we also looked for LN metastasis and tumor stage as our secondary endpoints.

Details of the three meta-analyses and study-specific findings are described in Tables 1-2.

**Table 1:** Description of the meta-analyses covered in the umbrella review PD-L1, programmed death-ligand 1. Table credit: Gashaw Hassen, Nidhi Jain, and Amita Kasar.

Study description	Meta-analysis 1	Meta-analysis 2	Meta-analysis 3
Author	Zhang et al., 2016 [18]	Gu et al., 2017 [19]	Qiu and Du, 2021 [20]
Link	https://www.nature.com/articles/srep37933	https://journals.plos.org/plosone/article?id=10.1371/journal.pone.0182692	https://jgo.amegroups.com/article/view/49395/html
Title	The clinicopathological and prognostic significance of PD-L1 expression in gastric cancer: a meta-analysis of 10 studies with 1,901 patients	PD-L1 and gastric cancer prognosis: a systematic review and meta-analysis	Clinicopathological and prognostic significance of PD-L1 expression in gastric cancer: a meta-analysis
Link for a table with a list of included studies	https://www.nature.com/articles/srep37933/tables/1	https://doi.org/10.1371/journal.pone.0182692.t001	https://europepmc.org/articles/PMC7944163/table/t1/
Total studies included (40)	10	15	15

**Table 2:** Summary of study and patient characteristics including prognostic factors TSS, total sample size; HR, hazard ratio; OR, odds ratio; LN, lymph node; NA, not available; PD-L1(+): PD-L1 presence/expression/positivity. *OS is described as five-year survival and TNM stage (III-IV vs I-II) as TNM stage (low vs high). Note: The study population includes those diagnosed with GC (Stage I-IV). Table credit: Gashaw Hassen, Nidhi Jain, and Amita Kasar.

Study and patient characteristics of the three meta-analyses included in the umbrella review					Outcomes/effect measures in PD-L1(+)		
					OS	Clinicopathological characteristics	
						LN metastasis	TNM stage (III-IV vs I-II)
					HR (95% CI)	OR (95% CI)	OR (95% CI)
Study author & publication year	Study period (14 years)	TSS (8419)	Male (%)	PD-L1(+) (%)			
Zhang et al., 2016 [18]	2006-2016	1901	38.9	39.5	1.64 (1.11-2.43)	2.17 (1.04-4.52)	2.36 (0.83-6.69)
Gu et al., 2017 [19]	2006-2017	3291	32.9	38.2	1.46 (1.08-1.98)	0.54 (0.31-0.95)	0.72 (0.40-1.28)
Qiu and Du, 2021 [20]*	2014-2019	3227	NA	39.9	1.39 (1.14-1.69)	1.73 (1.18-2.54)	1.28 (0.81-2.02)

Search Strategy and Selection Criteria

We performed an umbrella meta-analysis on previously published meta-analyses (studies) using Preferred Reporting Items for Systematic Reviews and Meta-Analyses (PRISMA) guidelines in August 2021. We used PubMed for finding out meta-analyses comparing the prognosis of GC patients based on the presence of PD-L1.

We used the following keywords: gastric cancer OR carcinoma OR GC and PD-L1 presence OR programmed death ligand 1 AND prognosis.

**Figure 1:**
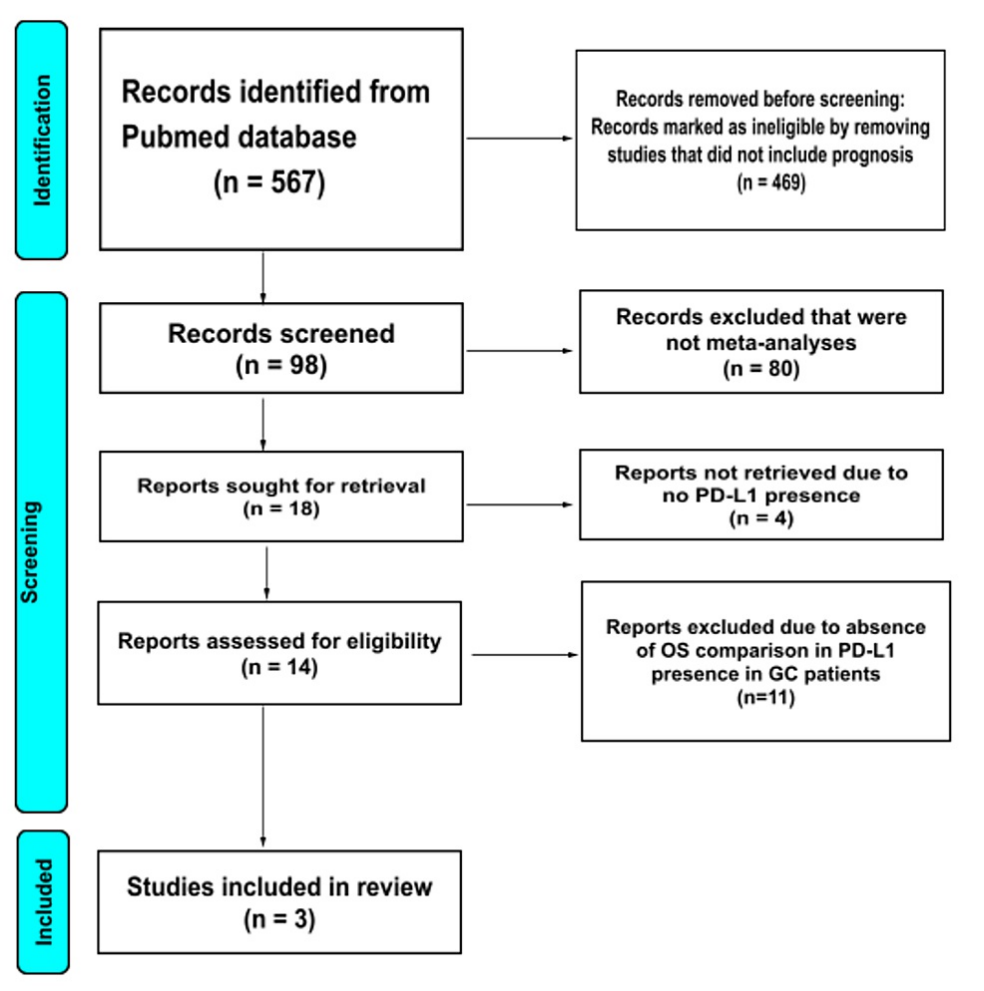
PRISMA 2020 flow chart of study selection PRISMA, Preferred Reporting Items for Systematic Reviews and Meta-Analyses. Figure credit: Urvish K. Patel and Nidhi Jain.

Inclusion/exclusion criteria: All published meta-analyses that compared the presence of PD-L1 as a prognostic indicator for GC patients were included. Any literature other than meta-analyses such as case reports, review articles, and observational studies were excluded. Non-English literature, non-full text articles, and non-human studies were excluded. The flow diagram of the study selection process is described in Figure 1.

Study Selection

All studies were identified using the search strategy described above and screened independently by four authors (Gashaw Hassen, Amita Kasar, Nidhi Jain, and Shivakshi Berry) for their eligibility, and any disagreement was resolved through discussion with senior authors (Urvish Patel and Rishabh Kumar Rana). Studies describing meta-analysis of observational studies were considered for full-text evaluation.

Data Extraction and Quality Assessment

A data extraction form on an excel sheet was used to extract data from the included studies for the assessment of literature synthesis. This form was designed in consultation with the biostatistician and methodologist in the team (Michlene Zouetr and Sheetal Kamat).

Extracted information included: study setting (study name, year of publication), study design, study population demographics (sex in %), sample size, GC stage and the presence of PD-L1 (in %), details of the PD-L1(+) primary outcomes such as overall/five-year survival rate, and other associated secondary outcomes such as tumor stage and LN metastasis. A table was used for information on the assessment of the risk of bias (using the Newcastle-Ottawa scale - NOS). Two review authors (Rishabh Kumar Rana and Sheetal Kamat) extracted the data independently, and the differences identified were resolved through discussion with other reviewers (Tejaswini Kurapati and Stephaine Oshai).

Statistical Analysis

We performed the analysis using Review Manager 5.3 software (RevMan, The Cochrane Collaboration; Stata Corp, College Station, USA). HR, OR, and 95% CI were mainly used for data analysis. HR > 1 in those with elevated PD-L1 expressions implied a worse prognosis. The Mantel-Haenszel formula was used to calculate dichotomous variables to obtain ORs with 95% CIs, which describes the outcome comparison between GC with PD-L1 expression vs GC with absent PD-L1. The inverse variance was used regardless of heterogeneity to estimate the combined effect and its precision as well as to give a more conservative estimate of the ORs and 95% CIs. To evaluate heterogeneity, we used *I*^2^ statistics and ≥75% was considered significant heterogeneity. A *P*-value <0.05 was considered statistically significant. Publication bias was assessed visually using funnel plots, and individual and overall study biases were described using the NOS (online supplemental file 1). The pooled OR and 95% CI are represented in the form of forest plots. Between-study heterogeneity (*I*^2^/*I*-square), evidence of small-study effects, and excess significance were assessed.

Results

Results of Literature Screening and Study Selection

A PubMed database search generated a total of 567 articles. Before the screening, 469 ineligible articles that did not address prognosis were removed, resulting in 98 records. On screening, 80 studies were excluded owing to non-conformity to meta-analysis, resulting in 18 reports sought for retrieval. Four studies were removed from further retrieval due to the absence of PD-L1. The remaining 14 studies were assessed for eligibility. Eleven studies were finally excluded for not assessing OS in PD-L1-positive GC. In the end, three meta-analyses were included for umbrella review with a total of 8,419 sample size. The screening procedure in the study selection process is shown in the flowchart, as depicted in Figure 1.

PD-L1 as a Prognostic Factor for GC Patients

**Figure 2:**
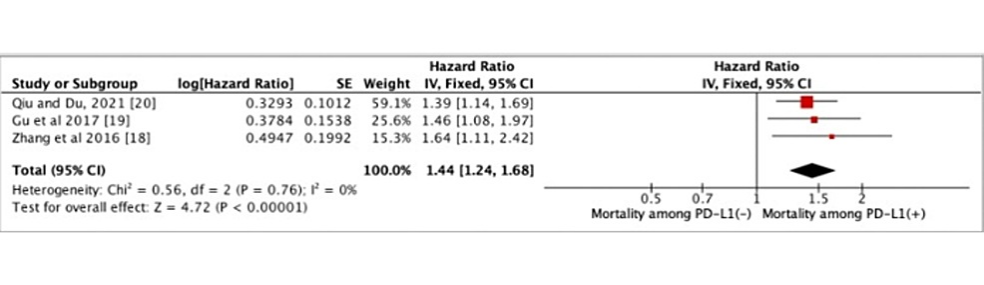
Forest plot describing PD-L1 expression and mortality among patients with GC CI, confidence interval; GC, gastric cancer; PD-L1, programmed death-ligand 1. Figure credit: Urvish K. Patel.

Our umbrella approach evaluated the correlation between PD-L1 expression and OS among the GC patients studied in the three meta-analyses. All three studies included in the umbrella review reported that positive expression of PD-L1 was associated with a poor prognosis of GC. The umbrella analysis showed that PD-L1-positive expression was associated with low OS in GC with increased mortality (HR=1.44, 95% CI: 1.24-1.68, *P*<0.00001) (Figure 2).

Correlation of PD-L1 Expression With Clinicopathological Characteristics

**Figure 3:**
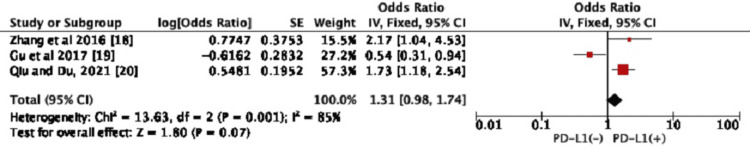
Forest plot for the association between PD-L1 expression and LN metastasis CI, confidence interval; LN, lymph node; PD-L1, programmed death-ligand 1. Figure credit: Urvish K. Patel.

**Figure 4:**
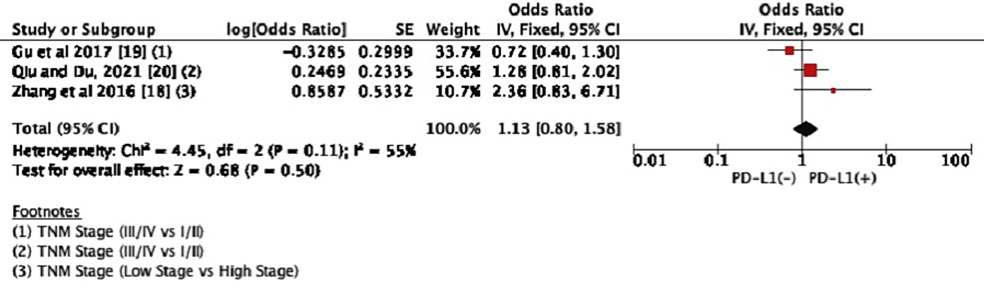
Forest plots for the association between PD-L1 expression and the TNM stage CI, confidence interval; PD-L1, programmed death-ligand 1; TNM, tumor, node, and metastasis. Figure credit: Urvish K. Patel.

We also investigated the correlation between PD-L1 expression and clinicopathological characteristics in GC, which included LN metastasis and the tumor, node, and metastasis (TNM) stage. Our umbrella analysis showed no significant correlation existed between PD-L1 expression and the clinicopathological features studied (Figures 3-4).

LN Metastasis

We performed an umbrella review of the two meta-analyses only by excluding the Gu et al., 2017 study to reduce heterogeneity, although the correlation between PD-L1 expression and lymphatic metastasis was reported in all three studies. Hence, the heterogeneity was reduced in the analysis of PD-L1 expression with LN metastasis (*P*=0.59; *I*^2^=0%). Then, the review results demonstrated that PD-L1 expression was not significantly related to LN metastasis. The positive expression of PD-L1 in the group with LN metastasis when compared to those without PD-L1 expression had no statistically significant difference (OR=1.31, 95% CI: 0.98-1.74, *P*=0.07) (Figure 3).

Tumor Stage

We finally analyzed the association between PD-L1 expression and the tumor stage which was reported in all three studies. We found no significant association between PD-L1 expression and the TNM stage (OR=1.13, 95% CI: 0.80-1.58, *P*=0.50) (Figure 4).

Sensitivity Analysis and Risk of Bias

**Figure 5:**
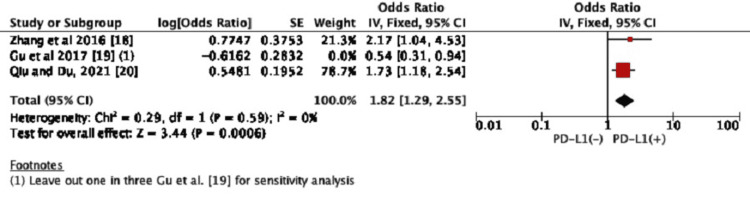
Sensitivity analysis of LN Metastasis and PD-L1 expression for heterogeneity. CI, confidence interval; LN, lymph node; PD-L1, programmed death-ligand 1. Figure credit: Urvish K. Patel.

**Figure 6:**
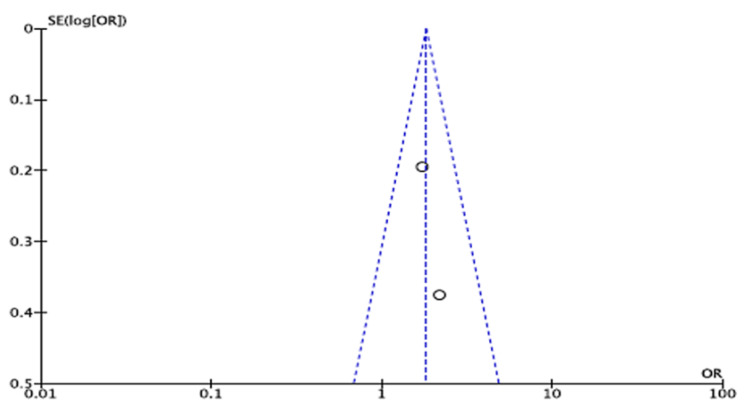
Funnel plot after sensitivity analysis using leave one out in the case of LN metastasis and the presence of PD-L1 in GC patients OR, odds ratio; LN, lymph node; PD-L1, programmed death-ligand 1. Figure credit: Urvish K. Patel.

**Figure 7:**
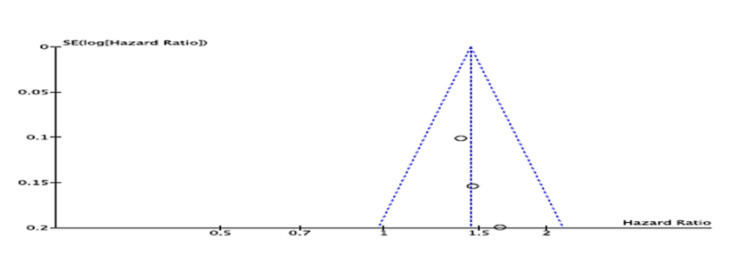
Funnel plot of PD-L1 presence and OS in GC patients OR, odds ratio; PD-L1, programmed death-ligand 1; GC, gastric cancer. Figure credit: Urvish K. Patel.

**Figure 8:**
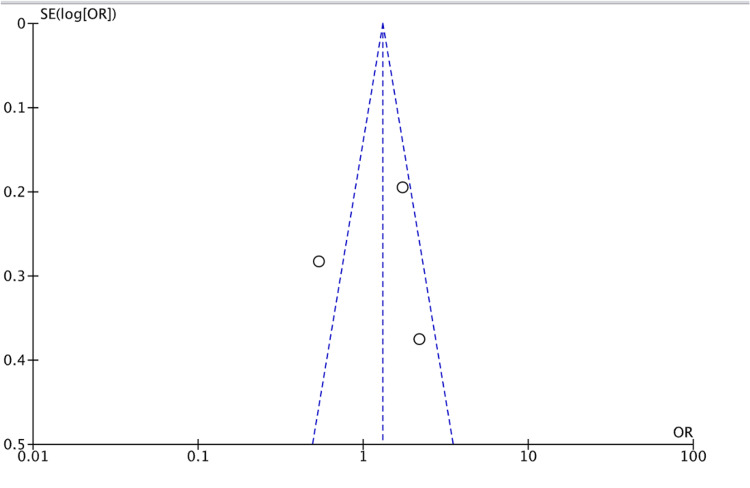
Funnel plot for LN metastasis and the presence of PD-L1 in GC patients OR, odds ratio; LN, lymph node; PD-L1, programmed death-ligand 1; GC, gastric cancer. Figure credit: Urvish K. Patel.

**Figure 9:**
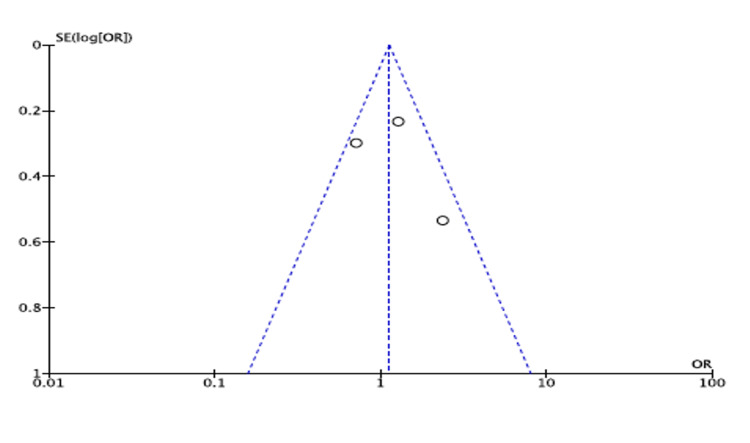
Funnel plot for TNM staging and PD-L1 in GC patients OR, odds ratio; TNM, tumor, node, and metastasis; PD-L1, programmed death-ligand 1; GC, gastric cancer. Figure credit: Urvish K. Patel.

We left one study out for sensitivity analysis and it revealed a significant change in overall heterogeneity regarding LN metastasis and PD-L1 presence, which indicated that the results had some bias (Figure 5). We also used NOS (Table 3) and funnel plots (Figures 6-9) for risk of bias assessment in the three meta-analyses (see appendices/supplementary files).

Discussion

In our umbrella review of three meta-analyses, the presence of PD-L1 in GC patients is associated with poor prognosis and reduced chances of OS. On the contrary, we could not find a statistically significant association of PD-L1 with LN invasion and TNM staging in patients with GC. The odds of LN invasion were not directly related to PD-L1 presence. Similarly, PD-L1 presence was not a good indicator for gauging the TNM stage in patients with GC.

However, Wen et al. revealed through their research that immune cell PD-L1, PD1 and CD8 and tumor cell PD-L1 gain independent prognostic values after adjustment of the TNM stage and that an Immunocore (Abington, UK) computed after a thorough evaluation of CD8+ T-cells and PD-L1/PD-1 can help segregate GC patients having the same stage into low-, medium-, or high-risk subcategories, thus helping to decide immunotherapy for treatment [21].

Recent discoveries have established human PD-L1 or B7-H1 to be a dominant ligand that is critical for antigen-specific T-cell response through PD-1-dependent immune suppression. Over the years, this fact has been targeted for treatment and clinical trials, suggesting that immunological checkpoint-focused therapy can improve survival in a variety of cancer patients including GC [22,23].

In our review, we had an average of 39% PD-L1 positivity in the GC patients studied at different stages. Recent evidence from “Checkmate 032”, an ongoing cohort study involving more than five countries including the United States, has also documented the presence of PD-L1 in 30-35% of its GC population. A detailed look into our meta-analyses will show a relatively wider range of PD-L1 presence among different GC study populations [24].

Age also has an important role to play in the overall prognosis of patients with GC in deciding whether treatment using PD-L1 checkpoint inhibitors will result in increased life expectancy or not. Younger patients have a lower risk of death if PD-L1 inhibitors were used. The mechanism regarding why and how age interacts with PD-L1 presence remains unclear. In our analyses, we had a wide age range in all studies from 18 to 60 years [25].

We established that the poor prognostic outcome of PD-L1 presence in GC was due to significantly reduced survival (HR 1.44, *P*<0.00001). Supporting our findings, research conducted by Tamura et al. on GC patients also revealed that the primary tumor’s PD-L1 expression was interlinked with tumor infiltration. Weak staining of tumor for PD-L1 also correlated with significantly improved OS, while strong staining corresponded to poor survival. The expression of PD-L1 qualified as an independent prognostic factor for stage II/III GC patients who went through a curative surgery. Finally, the study concluded that PD-L1 is a useful prognostic factor for considering immunotherapy with immune checkpoint inhibitors for advanced-stage GC [26].

PD-L1 was also found to be an independent prognostic factor for OS in GC patients, and a raised PD-L1/PD-1 expression was linked to a significantly improved outcome according to a study conducted by Boger et al. The study suggested that PD-L1/PD-1 expression may be used as a surrogate marker for GC patients with PD-L1 positivity, thereby guiding immunotherapy with immune checkpoint antibodies [27]. A study by Wu et al. reviewing 28 studies concluded the presence of PD-L1 as a prognostic indicator in the majority of solid tumors with poor prognosis [23].

Epithelial-mesenchymal transition (EMT) is key to the invasion and metastases of carcinoma, a process in which epithelial cells lose their cell polarities and cell-cell adhesion. Zinc finger E-box binding homeobox 1 (ZEB1) is one of the key factors regulating the EMT. The PD-L1 gene promoter region contains a ZEB1 binding site and its expression might be regulated by the ZEB1 transcription factor. PD-L1 is controlled with interferon (IFN-т) through a janus kinase-signal transducer and activator of transcription (JAK-STAT) signaling and EMT. PD-L1 regulates the immunity against the tumor cells by influencing the cytotoxic activity of the T lymphocyte. The mechanisms behind how exactly PD-L1 influences tumor immunology are still not very clear. Recently, a study by Ubukata et al. has demonstrated how PD-L1 is involved in tumor invasion with more certainty which, however, still needs more research to strengthen the evidence [28-30].

In our study, we had varying inferences from different studies and we could not establish a definitive association between LN metastasis and PD-L1 presence in GC patients.

The major strength of our research is a large sample size covering three meta-analyses with 8419 participants in 40 studies that give systematic evidence coalescing the latest scientific research on the association of PD-L1 with GC and its prognostic factors.

Some of the limitations of our study include publication bias (see funnel plots), dichotomous study results, geographical limitation, lack of standard definition for PD-L1 positivity, limited racial diversity, and heterogeneity in study designs.

## Conclusions

We can reiterate with a fair amount of certainty that PD-L1 positivity is associated with a poor prognosis in GC patients. The findings of this umbrella study will create opportunities in strengthening the clinicopathological understanding of GC. Our study will also bring insights among the scientific community, in general, and physicians, in particular, regarding the need for guided therapy tailored toward PD-L1-positive GC patients. More research and suitably designed studies are required to understand how PD-L1 positivity influences GC progress in terms of tumor invasion and LN metastasis.

## Appendices

**Table 3:** Risk of bias of included studies

Study	Newcastle-Ottawa scale (NOS)			
	Selection	Comparability	Outcome	The overall risk of bias
Zhang et al., 2016 [18]	**	*	**	Moderate
Gu et al., 2017 [19]	***	*	*	Low
Qiu and Du, 2021 [20]	***	*	**	Moderate
